# Synthesis of Porphyrin-Dendrimers with a Pyrene in the Periphery and Their Cubic Nonlinear Optical Properties

**DOI:** 10.3390/molecules16086950

**Published:** 2011-08-15

**Authors:** Eric G. Morales-Espinoza, Irina V. Lijanova, Omar G. Morales-Saavedra, Vícente Torres-Zuñiga, Simon Hernandez-Ortega, Marcos Martínez-García

**Affiliations:** 1 Instituto de Química, Universidad Nacional Autónoma de México, Cd. Universitaria, Circuito Exterior, Coyoacán, C.P., México D.F. 04510, Mexico; 2 Instituto Politécnico Nacional, CIITEC, Cerrada Cecati S/N, Colonia Santa Catarina de Azcapotzalco, C.P., México D.F. 02250, Mexico; 3 Centro de Ciencias Aplicadas y Desarrollo Tecnológico, Universidad Nacional Autónoma de México, CCADET-UNAM, Circuito exterior S/N, Ciudad Universitaria C.P., México D.F. 04510, Mexico

**Keywords:** pyrene, porphyrin, dendrimers, nonlinear optics

## Abstract

Dendrons of pyrene derivatives were attached to a porphyrin core. A marked effect in solution for the dendrimers was observed in the absorption spectra. All the compounds obtained were characterized by ^1^H-, ^13^C-NMR, FTIR, UV-vis, MALDI-TOF or FAB+ mass spectrometry and elemental analysis. The cubic nonlinear optical behavior of some the synthesized compounds was tested *via* Z-Scan measurements in spin-coated film samples.

## 1. Introduction

Dendrimers are highly branched polymers with a well defined structure, uniform size and molecular weight [1,2,3,4,5,6,7,8]. They are composed by a multifunctional central core to which dendritic branches are connected [9,10,11,12,13]. According to their shape, dendrimers are divided into two types. The first one has a circular or elliptic shape in which repeat units are regularly stretched from a core [14,15], and the second type has a conic shape with repeated units directionally stretched from a core [16,17,18]. Since it is possible to control the dendrimer’s size and structure, and at the same time introduce different kinds of functional groups into their terminal groups or core, numerous applications for these molecules have been found in different fields such as catalysis [19,20,21,22], biomedicine, energy or charge-transfer systems [23,24,25,26,27], sensors [28], charge transfer of light-emitting layer in organic light emitting diodes (OLEDs) [29,30,31], *etc.* In addition to the above advantages, dendrimers manifest a highly ordered structure and produce thin films with various functional groups on the top surface of the substrate, making possible the creation of unique materials in which surface characteristics are controlled at a molecular level. In the present work we report the synthesis of two generations of dendrimers with molecules of pyrene on the periphery and a tetraphenylporphyrin core. 

## 2. Results and Discussion

**Scheme 1:**
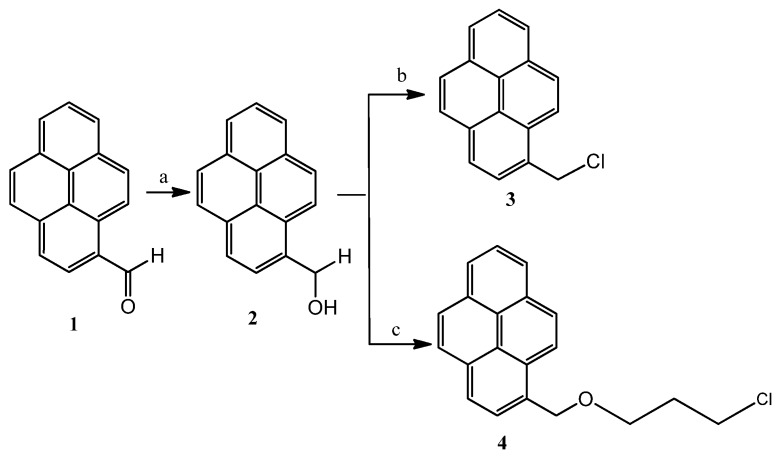
Synthesis of the pyrene derivatives.

The synthesis of the two pyrene-derivative dendrimer series was carried out applying a convergent pathway that consists of two steps, the first one being the synthesis of the two dendrons, and the second one the *O*-alkylation of the dendrons to the porphyrin. Following this order, dendrons containing pyrenebenzyloxy- and pyrene-1-methyl-3-chloropropoxybenzyloxy groups were prepared according to the convergent Fréchet approach [32] starting from a reduction reaction from the commercially available 1-pyrenecarboxaldehyde **1** via the reduction of the aldehyde **1** to obtain the pyren-1-ylmethanol **2** and its chlorination with SOCl_2_ and pyridine to obtain compound **3** (Scheme 1) or its *O*-alkylation with 1-bromo-3-chloropropane in acetone and K_2_CO_3_ at reflux to obtain the compound **4** (Scheme 1).

**Figure 1:**
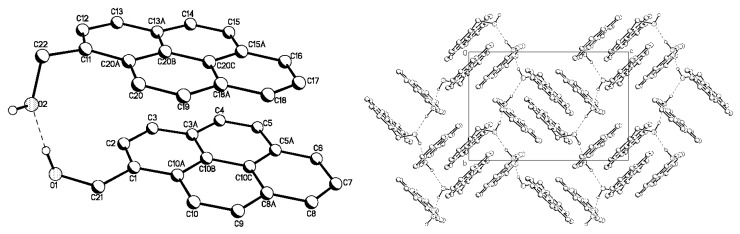
Crystal structure and crystal packing of the 1-(chloromethyl)pyrene **2**. Selected bond lengths [Å]: O(1)-C(21) = 1.421, C(21)-C(1) = 1.508, C(1)-C(2) = 1.378. Selected angles (°): O(1)-C(21)-C(1) = 114.2, C(21)-C(1)-C(2) = 120.2, C(1)-C(2)-C(3) = 121.9.

The following signals were observed in the ^1^H-NMR spectrum of compound **2**: one singlet at δ_H_ 1.83 for the OH proton, one singlet at δ_H_ 5.36 due to the CH_2_ group and for the pyrene group (multiplets at δ_H_ 7.96–8.34). The structure of the pyren-1-ylmethanol compound **2** was confirmed by X-ray diffraction studies (Figure 1). In the crystal structure, a hydrogen bridge was observed between two pyren-1-ylmethanol moieties and the crystal packing of compound **2** showed strong interactions between the four pyrene molecules.

The chemical structure of the 1-(chloromethyl)pyrene **3** was confirmed by ^13^C-NMR spectroscopy. In the spectrum, the most significant resonance signals were those observed at δc 44.77 of the CH_2_-Cl group, and the aromatic signals due to the pyrene carbons.

In the ^1^H-NMR spectrum of compound **4**, the following signals were observed: one multiplet at δ 2.03 due to the CH_2_ group, two multiplets at δ 3.63 and at δ 4.09 assigned to the CH_2_-Cl and CH_2_-O groups with a coupling constants *J* = 6.4 and *J* = 1.6 Hz, respectively. One singlet at δ 5.12 due to the CH_2_-O joined to the pyrene, and finally the characteristic signals due to the pyrene moiety. The structure of the compound **4** was further confirmed by mass spectrometry.

**Scheme 2:**
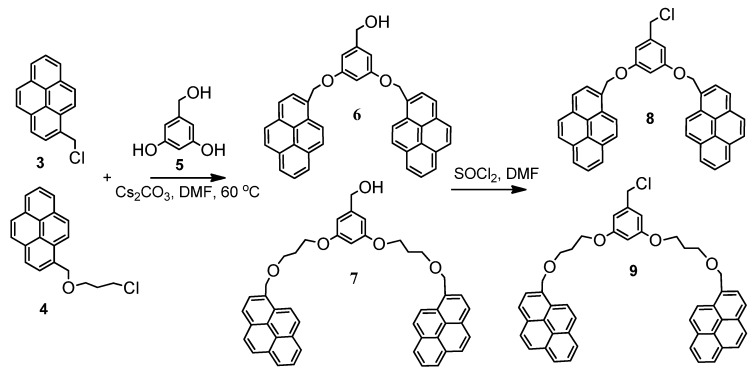

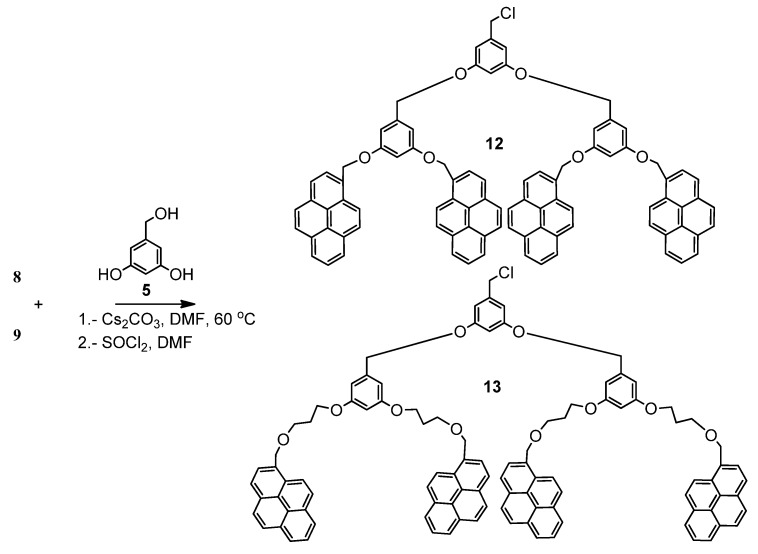
Synthesis of dendrons **8**, **9** and **12**, **13**.

Chlorides **3** and **4** were attached to the 3,5-dihydroxybencyl alcohol **5** in DMF, cesium carbonate at 60 °C to give alcohol intermediates **6**, **7**, which were converted into chlorides **8** and **9** upon treatment with thionyl chloride and pyridine in dichloromethane (Scheme 2). Dendrons **8** and **9** are the first generation dendrons of the pyrene derivative families. 

The second generation dendrons **12** and **13** were obtained from the dendrons **8** and **9** following the same methodology. First they were attached to 3,5-dihydroxybenzyl alcohol (**5**) in DMF using cesium carbonate at 60 °C to give alcohols **10**, **11**, which were converted into chlorides **12** and **13** upon treatment with thionyl chloride and pyridine in dichloromethane. Dendrons **12** and **13** are the second generation dendrons of the pyrene derivatives families.

The structures of **12** and **13** was confirmed by ^1^H-NMR spectroscopy. Signals at δ_H_ 4.60, 5.76 (dendron **12**) and δ_H_ 4.35, 5.15 (dendron **13**) corresponded to the methylene protons Ar-CH_2_-Cl and Pyrene-CH_2_-O. Compound **13** showed three signals at δ_H_ 2.03, 3.73 and 4.59 due to the propyloxy groups. The aromatic protons were observed as one singlet at δ_H_ 6.84 and 6.80, for compound **12** and **13**, respectively. The characteristic signals for the pyrene moiety also were observed.

**Scheme 3:**
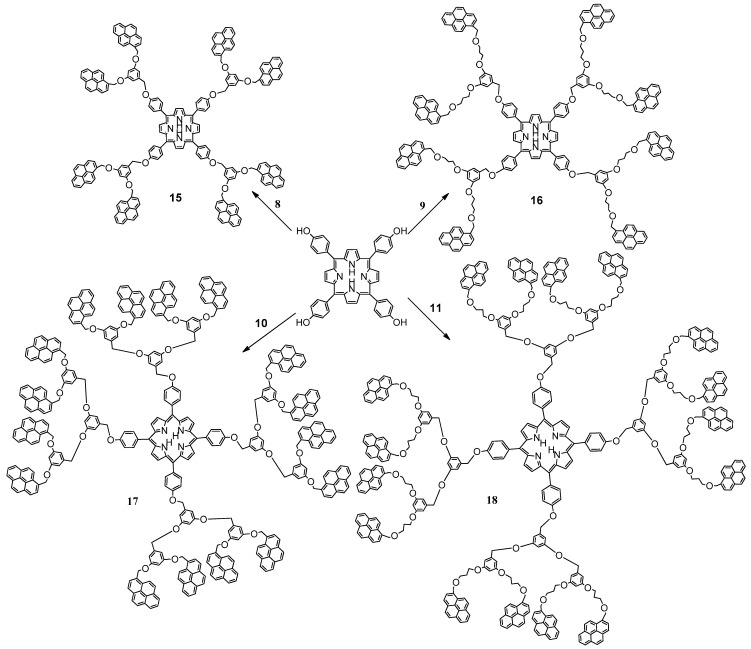
Synthesis of dendrimers **15**–**18**.

The synthesis of the dendrimers is depicted in Scheme 3. This synthesis involves only one step, an O-alkylation between dendrons **8**, **12** or **9**, **13** and porphyrin **14**. The reaction was carried out in dimetylformamide and Cs_2_CO_3_ at 60 °C for 3 days and the dendrimers were obtained in good yields.

**Figure 2:**
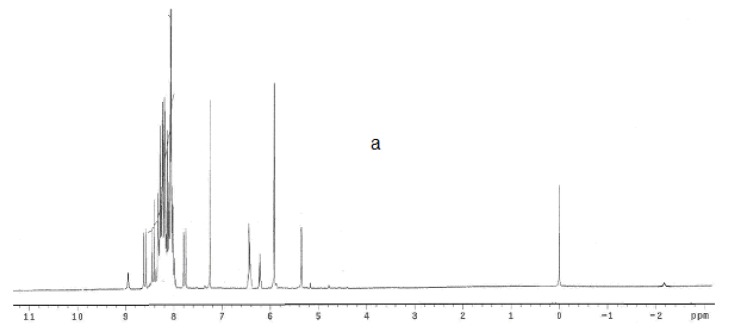

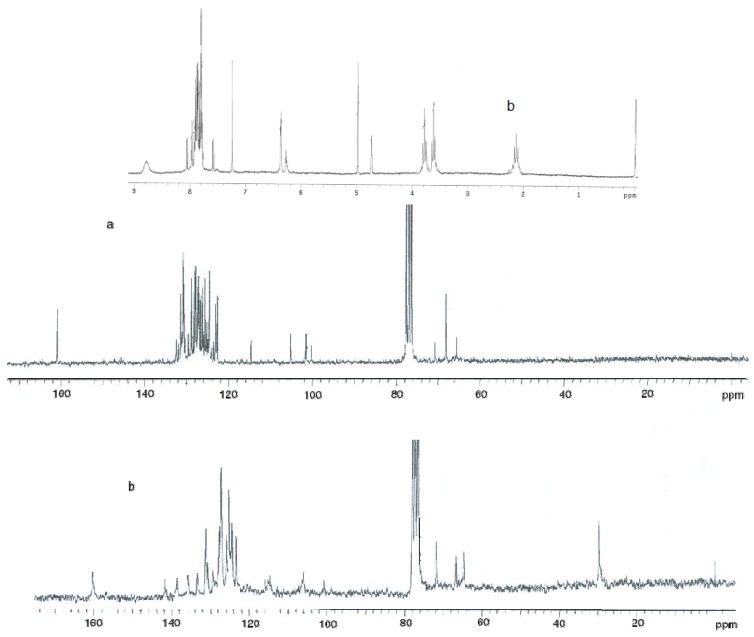
^1^H-NMR spectra of dendrimers **15** (a) and **16** (b) in CDCl_3_ at room temperature.

The ^1^H-NMR spectra of the dendrimers **15** and **16** (Figure 2) showed one broad signal at δ_H_ −2.23 due to the N-H protons, three multiplets at δ_H_ 2.17, 3.57 and 3.97 assigned to the CH_2_ protons of the propyl group, two singlets at δ_H_ 4.77 and δ_H_ 4.98 due to the Ar-CH_2_-O and Pyrene-CH_2_-O. The aromatic protons at the dendron were observed as two broad signals at δ_H_ 6.25 and δ_H_ 6.36. The characteristic signals for the pyrene moiety at δ_H_ 7.74 to δ_H_ 8.02 were also observed. Two doublets at δ_H_ 7.56 and 8.07 were assigned to the aromatic protons with a coupling constant of *J* = 8.2 Hz joined to the porphyrin. Finally, one broad signal at δ_H_ 8.79 was assigned to the pyrrole ring. 

The structures of dendrimers **15**–**18** were also confirmed by ^13^C-NMR, IR and MALDI-TOF mass spectrometry. It was found that all the dendrimers have the expected molecular weight. 

**Figure 3:**
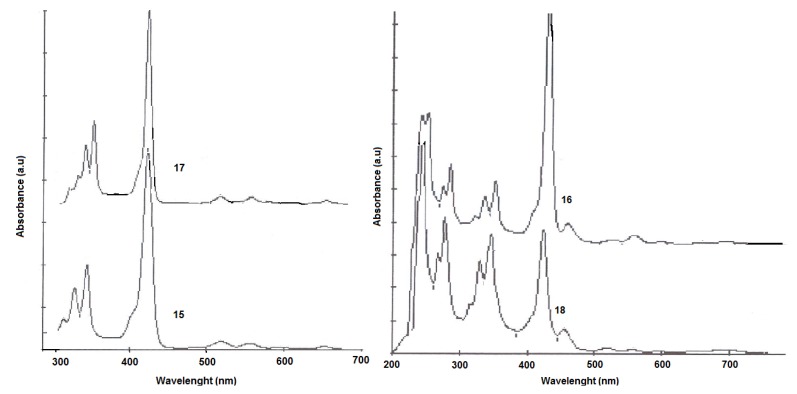
UV-vis spectra of dendrimers **15**–**18** in DMF at 1 × 10^−6^ M.

In the optical absorption spectra of the dendrimers **15**–**18** (Figure 3) in two different solvents, CH_2_Cl_2_ and dimethylformamide (DMF), both Soret and Q-bands do not shift upon increasing the concentration up to 10^−6^ M, indicating the absence of intermolecular aggregation processes in the ground state. Bands at 329 and 345 nm due to the pyrene molecules and in the case of dendrimers **16** and **18**, bands were observed at 243, 277, 328 and 344 nm. The tetraphenylporphyrin clearly shows an intense band at 423 nm and for the dendrimers **16** and **18**, new high energy bands were observed at 453 and at 689 nm, along with the Q- bands (see Table 1).

### 2.1. Linear and Third Order Non-Linear Optical Characterization

Regarding the linear and nonlinear optical properties of the obtained materials, homogeneous film samples were successfully deposited on glass substrates via spin-coating procedures from first and second generation dendrons (compounds **9** and **13**, respectively), and from their respective dendrimer systems (compounds **16** and **18**, respectively). Selected samples were chosen for cubic NLO-characterization *via* the Z-Scan technique.

**Figure 4:**
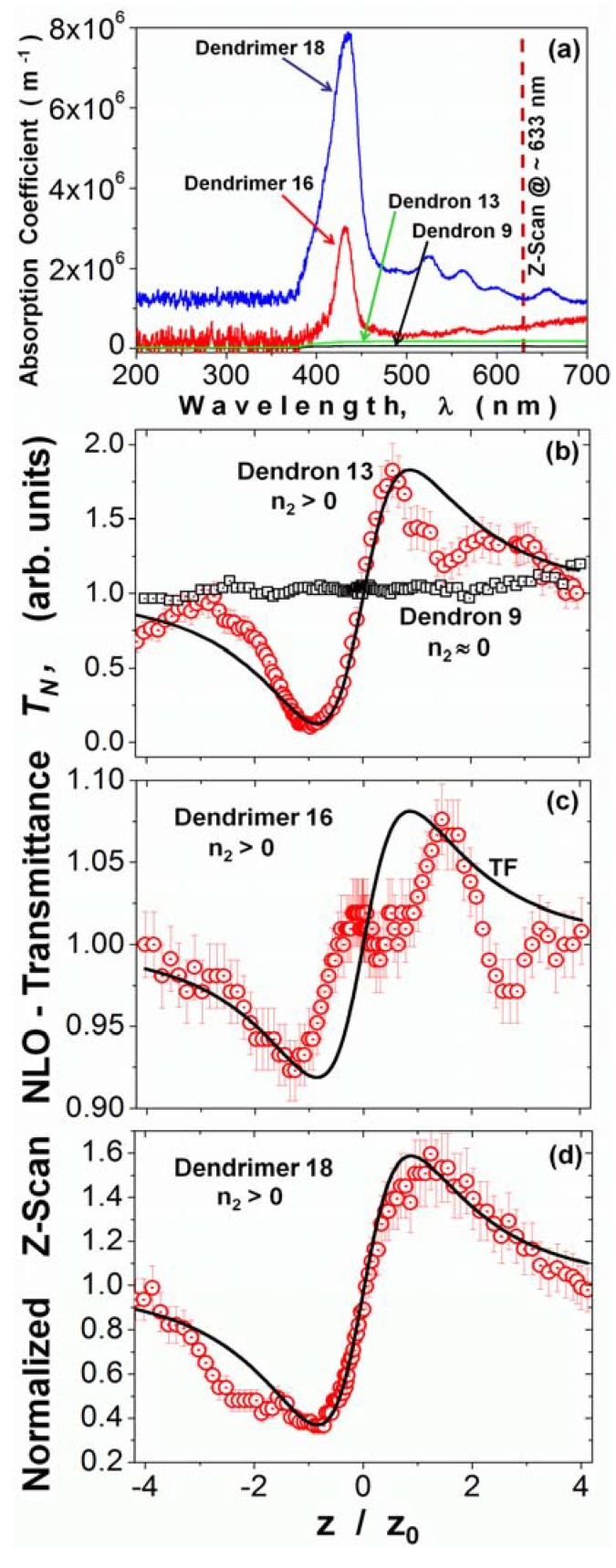
Linear and nonlinear optical measurements obtained for the pristine reference dendrons (**9** and **13**) and the dendrimer-based film samples (**16** and **18**): (**a**) Linear absorption coefficients of the film samples evaluated within the visible range; (**b**) Closed aperture Z-Scan data obtained at λ_Z-Scan_ = 632.8 nm for the first and second generation dendron-based films; and (**c**-**d**) closed aperture Z-Scan data obtained for the dendrimer-based films under similar experimental conditions. An estimated experimental error below 5% is also considered for the Z-Scan data (error bars). Theoretical fitting: continuous lines.

Figure 4a shows the linear absorption coefficients evaluated within the visible range for the deposited samples. Indeed, the Beer-Lambert law applies for such semi-transparent films allowing an adequate data analysis and making these materials potential candidates for optical applications due to their appropriate transparency. It is evident from Figure 4a that the highest absorptive properties occur within the 370–550 nm spectral range, which may indicate additional conjugation of delocalized π-electrons and multi-directional charge transfer provided by the higher content of dendrons within the dendrimer-based film samples. This suggestion will be explored by means of NLO Z-Scan experiments as explained below. Under this framework, the available laser excitation line for Z-Scan experiments (λ_Z−Scan_ = 632.8 nm) is also depicted in this figure (vertical dashed line). At this wavelength, low absorption conditions occur, allowing non-resonant NLO-characterizations of the samples which are a critical point when working with photodegradable organic materials. In fact, relatively small linear absorption coefficients (see Table 2) were evaluated for the studied samples at λ_Z-Scan_. These values are very useful for the determination of the nonlinear refraction and absorption coefficients according to the Z-Scan technique. The thicknesses and estimated linear refractive indices of the studied samples are also shown in Table 2.

Z-Scan measurements were performed at room conditions on the developed films. The observed non-local effect of these samples is shown in Figures 4(b-d). A rigorous theoretical fitting was performed in order to evaluate both the nonlinear absorptive and refractive properties of these samples. The NLO-response of the developed dendron- and dendrimer-based films was characterized by varying the polarization input planes of the He-Ne laser system in order to explore microscopic material asymmetries or anisotropies throughout the sample structure. In general, since all NLO-measurements were systematically performed with different laser input polarization states (from 0 to 90°: *s*- to *p*-polarization, respectively) and the obtained curves are quite similar in each sample, the film structures do not seem to show any significant anisotropic behaviour, thus confirming their amorphous nature. As shown in Figure 4(b) and Table 2, the Z-Scan curves obtained from the pristine, highly transparent dendron-based films (compounds **9** and **13**), exhibit negligible nonlinear refraction and absorption when compared to the curves obtained for the dendrimer films under the same laser power regime. Indeed, compound **9** shows a flat Z-Scan curve and only compound **13** exhibits a typical valley-to-peak Z-Scan transmittance curve. Taking into account the theory developed by Sheik-Bahae *et al*. and Liu *et al*. [33,34,35,36,37,38] it is observed from our measurements that the nonlinear refractive response of the studied samples can be unambiguously determined by these typical Z-Scan transmittance curves. Hence, one can immediately observe that the dendron (**13**) and dendrimer-based films exhibit positive NLO-refraction coefficients (γ or *n_2_* > 0).

The respective theoretical fits (TFs) to the obtained Z-Scan transmission data (solid lines) are also shown in Figures 4(b-d). In order to perform TFs according to previous theoretical models, the normalized Z-Scan transmittance (*T_N_*) can be determined as a function of the dimensionless sample position (*x = z/z_0_*)_,_ where z_0_ is the Rayleigh range and z is the Z-Scan sample position. Hence, the TFs were obtained according to the following equation, considering both nonlinear refraction and absorption effects [38]:

*T_N_* ≈ 1+[4*x*/(1+*x*^2^)(9+*x*^2^)]ΔΦ−[2(*x*^2^+3)/(1+*x*^2^)(9+*x*^2^)]ΔΨ (1)

Here, the first term is related to NLO-refractive effects whereas the second one is associated to NLO-absorptive phenomena. Indeed, since some of the obtained Z-Scan curves clearly exhibit a valley-to-peak transmittance asymmetry, NLO absorption effects are also expected [16]. The fitting parameters are in this case the induced phase shifts ΔΦ or ΔΨ, respectively. In the former case, the phase shift is given by ΔΦ=2*πγI*_0_*L_eff_*/*λ*, [38] from which the NLO refractive index (γ- coefficient) can be obtained. In the latter case, the phase shift is provoked by the NLO-absorption and is given by ΔΨ=*βI*_0_*L_Eff_*/2, [38] allowing the evaluation of the NLO absorption (*β*-coefficient), either due to two photon (TPA) or multi-photon absorption (*β* > 0), and/or saturable absorption (*β* < 0). In these equations, λ is the laser wavelength, *I_0_* is the input beam intensity (at focal spot: *z* = 0) and *L_eff_* is the effective thickness of the film sample, defined as *L_eff_* = ⎾1−(*e*^−*α*_0_*Ls*^)⏋(*α*_0_)^−1^, where *α*_0_ represents the linear absorption coefficient. All these equations are well established and have been proven in early Z-Scan works [33,34,35,36,37,38]. The theoretical restrictions imposed by these formulas in order to apply such expressions at optimal conditions (|ΔΦ_0_| < *π*, S ≈ 20%, *etc.*), are not always fully satisfied in our experimental results due to the large phase shifts and huge nonlinearities. Nevertheless, in most cases (mainly in the case of well defined γ > 0 or γ < 0 curves); our results nearly satisfy these conditions and can be conveniently fitted according to these theoretical formulas. Thus, for comparison purposes and in order to be consistent with the estimation of the γ- and β-values, we assumed their applicability and used them in our experimental results. According to Table 1 the TFs allowed us to evaluate a positive NLO-refractive coefficient in the order of γ = +0.684 × 10^−8^ m^2^W^−1^ (or n_2_ = +2.56 × 10^−2^ esu) for the dendron-**13** based film sample. In contrast, NLO-refractive coefficients as high as n_2_ = +9.27 × 10^−2^ esu and n_2_ = +104 × 10^−2^ esu were evaluated for the dendrimer-based films (compounds **16** and **18**, respectively). The obtained γ/n_2_-values are very large, many orders of magnitude larger than those observed for typical glass substrates or for the classical CS_2_ standard reference material: +1.2 × 10^−11^ esu (Z-Scan at λ = 10.6 μm) or 6.8 × 10^−13^ esu (DFWM at λ = 532 nm) [33,35].

On the other hand, only compounds **13** and **18** exhibited measurable NLO absorption at the implemented experimental conditions, where the positive sign obtained for the β-coefficients reveal the nature of the NLO absorptive phenomena in these -second generation- based samples; indicating strong multi-photon absorption effects [33,34,35,36,37,38]. This fact points to the onset of thermal effects during Z-Scan experiments due to long cw-laser irradiation. Indeed, Z-Scan experiments were performed at low laser energy conditions (~6.6 mW) in order to avoid as much as possible photodegradation of the samples.

### 2.2. Crystal Structure Determination

A suitable crystal of compound **2** (obtained by crystallization from CH_2_Cl_2_ at room temperature) was rolled in epoxy resin and mounted on a glass fiber. Bruker Apex AXS CCD area detector X-Ray diffractometer was the instrument used for the determination. The data were first reduced and corrected for absorption using psi-scans, and then solved using the program SHELL-XS. All nonhydrogen atoms were refined with anisotropic thermal parameters and the hydrogen atoms were refined at calculated positions with thermal parameters constrained to the carbon atom on which they were attached. A summary of the key crystallographic information is given in Table 3. CCDC 838806 contains the supplementary crystallographic data for this paper. These data can be obtained free of charge via www.ccdc.cam.ac.uk/conts/retrieving.html (or from the CCDC, 12 Union Road, Cambridge CB2 1EZ, UK; Fax: +44 1223 336033; E-Mail: deposit@ccdc.cam.ac.uk)

## 3. Experimental

### 3.1. General

Solvents and reagents were purchased as reagent grade and used without further purification. Acetone was distilled over calcium chloride. Tetrahydrofuran was distilled from sodium and benzophenone. Column chromatography was performed on Merck silica gel 60Å (70–230 mesh). ^1^H- and ^13^C-NMR were recorded on a Varian-Unity-200 MHz with tetramethylsilane (TMS) as an internal reference. Infrared (IR) spectra were measured on a spectrophotometer Nicolet FT-SSX. Elemental analysis was determined by Galbraith Laboratories, Inc. (Knoxville, TN, USA). FAB+ mass spectra were taken on a JEOL JMS AX505 HA instrument. MALDI-TOF mass spectra were taken on a Bruker Omni FLEX instrument.

### 3.2. Synthesis of Dendrons and Dendrimers

*1-Pyrenemethanol* (**2**): 97% Lithium aluminum hydride (1.7 mmol) was dissolved in dry THF (50 mL). To this solution, compound **1** (6 mmol) dissolved in dry THF (15 mL) was added dropwise using an addition funnel. The reaction was carried at 0 °C for 4 h. After this time, water (10 mL) was added and the reaction mixture was filtered through Celite^®^. The solvent was evaporated and the residue was dissolved in dichloromethane. The resulting solution was dried with sodium sulfate, filtered and the product was vacuum dried and purified by column chromatography (Al_2_O_3_, hexane), as a yellow powder, yield 5.82 mmol (97%). m.p. 123–125 °C. FTIR (KBr pellet, cm^−1^): 3278, 1594, 1180, 1064, 1008, 842, 705. UV-vis (CHCl_3_, nm) λ_max_: 344, 328, 314, 277, 266, 252, 243. ^1^H-NMR (CDCl_3_), δ (ppm): 1.83 (s, 1H, OH), 5.36 (s, 2H, Py-CH_2_-O), 7.96-8.34 (m, 9H, H_Py_). ^13^C-NMR (CDCl_3_), δ (ppm): 63.8 (CH_2_), 122.9 (C_Py_), 124.6 (C_Py_), 124.7 (C_Py_), 124.9 (C_Py_), 125.2 (C_Py_), 125.9 (C_Py_), 127.3 (C_Py_), 127.4 (C_Py_), 127.8 (C_Py_), 128.8 (C_Py_), 130.7 (C_Py_), 131.2 (C_Py_), 133.7 (C_ipso_). MS (*m/z*): 232 [M]^+^. Calc. for C_17_H_12_O: C, 87.90; H, 5.21 (%); Found: C, 87.91; H, 5.20 (%).

*1-Chloromethylpyrene* (**3**): A solution of pyridine (6.1 mmol) and SOCl_2_ (6.1 mmol) in dry CH_2_Cl_2_ (50 mL) was added to **2** (6 mmol), and then this mixture was cooled to −10 °C. The reaction was carried out in nitrogen atmosphere for 7 h. After this, the solvent was evaporated and the resulting oil was dried and purified by silica gel chromatography using a mixture of hexane-dichloromethane 2:1 as an eluent to afford 1-chloromethylpyrene (**3**) as a brownish powder, yield 5.76 mmol (96%). m.p. 148–149 °C. FTIR (KBr pellet, cm^−1^): 3423, 3042, 1593, 1253, 1182, 841, 757, 689. UV-vis (CHCl_3_, nm) λ_max_: 349, 339, 332, 279, 272, 268, 254, 244. ^1^H-NMR (CDCl_3_) δ (ppm): 5.25 (s, 2H, Py-CH_2_-Cl), 7.97–8.19 (m, 9H, H_pyr_). ^13^C-NMR (CDCl_3_) δ (ppm): 44.7 (CH_2_), 122.7 (C_Py_), 124.5 (C_Py_), 124.7 (C_Py_), 125.0 (C_Py_), 125.6 (C_Py_), 126.1 (C_Py_), 127.2 (C_Py_), 127.6 (C_Py_), 127.9 (C_Py_), 128.3 (C_Py_), 129.1 (C_Py_), 130.2 (C_Py_), 130.6 (C_Py_), 131.1 (C_Py_), 131.9 (C_ipso_). MS (*m/z*): 250 [M]^+^. Calc. for C_17_H_11_Cl: C, 81.44; H, 4.42; Cl, 14.14 (%); found: C, 81.44; H, 4.42(%).

*1-((3-Chloropropoxy)methyl)pyrene* (**4**): A mixture of 1-pyrenemethanol (**2**, 6 mmol), potassium carbonate (18 mmol) in dry acetone (100 mL) was heated to reflux and stirred vigorously in nitrogen atmosphere for 30 min. Then, 1-bromo-3-chloro propane (6.1 mmol) dissolved in dry acetone (40 mL) was added dropwise and the reaction was continued for 7 days. The mixture was allowed to cool and the precipitate was filtered. The filtrate was evaporated to dryness under reduced pressure. The residue, dissolved in diethyl ether, was washed with an aqueous solution of 5% Na_2_CO_3_ (three times). The organic layer was dried and evaporated to dryness and purified by silica gel chromatography using a mixture of hexane-dichloromethane 2:1 as an eluent to afford 1-[(3-chloropropoxy)methyl]pyrene (**4)**, as a yellow oil, yielding 2.34 mmol (39%). FTIR (KBr pellet, cm^−1^): 3042, 2859, 1077, 845, 708. UV-vis (CHCl_3_, nm) λ_max_: 341, 332, 325, 316, 312, 275, 269, 264, 250, 241, 214, 204. ^1^H-NMR (CDCl_3_) δ (ppm): 2.03 (m, 2H, CH_2_), 3.63 (m, 2H, Ar-CH_2_-Cl, *J* = 6.4 Hz), 4.09 (m, 2H, Ar-CH_2_-O, *J* = 1.6 Hz), 5.12 (s, 2H, Py-CH_2_-O), 7.90–8.32 (m, 9H, Ar-H). ^13^C-NMR (CDCl_3_) δ (ppm): 32.7 (CH_2_), 42.0 (CH_2_-Cl), 66.6 (CH_2_-O), 71.2 (Py-CH_2_-O), 117.3 (C_Py_), 122.3 (C_Py_), 124.4 (C_Py_), 124.6 (C_Py_), 124.8 (C_Py_), 125.1 (C_Py_), 125.8 (C_Py_), 126.8 (C_Py_), 127.3 (C_Py_), 127.6 (C_Py_), 129.2 (C_Py_), 130.7 (C_Py_), 131.1 (C_Py_), 131.2 (C_Py_), 131.3 (C_Py_), 134.7 (C_ipso_). MS (*m/z*): 308 [M]^+^. Calc. for C_20_H_17_ClO: C, 77.79; H, 5.55 (%); found: C, 77.77; H, 5.57 (%).

#### 3.2.1. General Procedure

A mixture of **3**, **4**, **8** or **9** (6 mmol) and cesium carbonate (12 mmol) in DMF (20 mL) was stirred vigorously in nitrogen atmosphere for 30 min. 3,5-Dihydroxybenzyl alcohol (**5**, 2.9 mmol) dissolved in DMF (10 mL) was added dropwise and the reaction was continued for 3 days. The mixture was filtered. The filtrate was evaporated to dryness under reduced pressure. The organic layer was dried and evaporated to dryness and purified by silica gel chromatography using a mixture of hexane-dichloromethane 2:1 as an eluent.

*(3,5-bis(Pyren-1-ylmethoxy)phenyl)methanol* (**6**): Yellow powder, yield 1.11 mmol (38 %). FTIR (KBr pellet, cm^−1^): 3430, 3039, 1593, 1448, 1352, 1287, 1165, 1021, 842, 707. UV-vis (CHCl_3_, nm) λ_max_: 344, 336, 328, 319, 314, 294, 277, 271, 267, 253, 244. ^1^H-NMR (CDCl_3_), δ (ppm): 1.62 (s, 1H, OH), 4.69 (s, 2H, Ar-CH_2_-OH), 5.72 (s, 4H, Py-CH_2_-O), 6.81 (s, 3H, Ar), 7.97–8.29 (m, 18H, H_Py_). ^13^C-NMR (CDCl_3_), δ (ppm): 65.3 (Ar-CH_2_-O), 68.8 (Py-CH_2_-O), 101.4 (C_Ar_), 106.0 (C_Ar_), 122.9 (C_Py_), 124.6 (C_Py_), 124.9 (C_Py_), 125.4 (C_Py_), 126.0 (C_Py_), 126.8 (C_Py_), 127.3 (C_Py_), 127.6 (C_Py_), 128.0 (C_Py_), 129.2 (C_Py_), 129.5 (C_Py_), 130.7 (C_Py_), 131.1 (C_Py_), 131.5 (C_ipso_Py ), 143.5 (C_ipso_ Ar), 160.3 (C_ipso_ Ar). MS (*m/z*): 568 [M]^+^. Calc. for C_41_H_28_O_3_: C, 86.60; H, 4.96 (%); Found: C, 86.60; H, 4.98 (%).

*(3,5-bis(3-(pyren-1-ylmethoxy)propoxy)phenyl)methanol* (**7**): Yellow powder, yield 0.72 mmol (25%). FTIR (KBr pellet, cm^−1^): 3350, 3038, 2946, 2857, 1599, 1497, 1165, 1088, 1054, 844, 704. UV-vis (CH_3_OH, nm) λ_max_: 341, 333, 326, 292, 275, 251, 241, 217, 207. ^1^H-NMR (CDCl_3_) δ (ppm): 1.68 (br, 1H, OH), 2.03 (m, 4H, CH_2_), 3.73 (t, 4H, CH_2_-O, *J* = 6.0 Hz), 3.90 (t, 4H, CH_2_-O, *J* = 6.0 Hz), 4.35 (s, 2H, Ar-CH_2_-O), 5.15 (s, 4H, Py-CH_2_-O), 6.11 (t, 1H, Ar, *J* = 2.2 Hz), 6.21 (d, 2H, Ar, *J* = 2.2 Hz), 7.88–8.27 (m, 18H, Py). ^13^C-NMR (CDCl_3_) δ (ppm): 29.6 (CH_2_), 64.4 (CH_2_-O), 65.1 (CH_2_-O), 66.4 (Ar-CH_2_-O), 71.6 (Py-CH_2_-O), 100.0 (C_Ar_), 104.7 (C_Ar_), 123.4–130.7 (C_py_), 131.3 (C_ipso_ Py), 142.8 (C_ipso_ Ar), 160.0 (C_ipso_ Ar). MS (*m/z*): 684 (M). Calc. for C_47_H_40_O_5_: C, 82.43; H, 5.89 (%); found: C, 82.45; H, 5.85 (%).

*(3,5-bis((3,5-bis(Pyren-1-ylmethoxy)benzyl)oxy)phenyl)methanol* (**10**): Yellow powder, yield 1.02 mmol (35%). FTIR (KBr pellet, cm^−1^): 3438, 2958, 2926, 2856, 1732, 1596, 1462, 1271, 1160, 845, 738, 707. UV-vis (CHCl_3_, nm) λ_max_: 346, 330, 315, 278, 269, 244. ^1^H-NMR (CDCl_3_), δ (ppm): 2.19 (s, 1H, OH), 4.58 (s, 2H, Ar-CH_2_-OH), 5.03 (s, 4H, Ar-CH_2_-O), 5.75 (s, 8H, Py-CH_2_-O), 6.85 (m, 9H, Ar), 7.97–8.32 (m, 36H, H_Py_). ^13^C-NMR (CDCl_3_), δ (ppm): 64.5 (Ar-CH_2_-O), 68.1 (CH_2_-O-Ar), 70.4 (Py-CH_2_-O), 100.1 (HC_Ar_), 101.8 (HC_Ar_), 105.0 (HC_Ar_), 106.5 (HC_Ar_), 122.9–130.8 (C_py_), 131.1 (C_ipso_ Py), 131.2 (C_ipso_), 132.2 (C_ipso_), 138.2 (C_ipso_), 141.33 (C_ipso_), 160.3 (C_ipso_). MALDI-TOF MS (*m/z*): 1240 (M). Calc. for C_89_H_60_O_7_: C, 86.11; H, 4.87 (%); found: C, 86.12; H, 4.85 (%).

*(3,5-bis((3,5-bis(3-(Pyren-1-ylmethoxy)propoxy)benzyl)oxy)phenyl)methanol* (**11**): White powder, yield 1.44 mmol (49%). FTIR (KBr pellet, cm^−1^): 3421, 3008, 2957, 2926, 2870, 1729, 1597, 1164, 1078, 847, 756, 708. UV-vis (CHCl_3_, nm) λ_max_: 344, 328, 316, 277, 266, 245. ^1^H-NMR (CDCl_3_) δ (ppm): 2.16 (s, 1H, OH), 2.03 (m, 8H, CH_2_), 3.78 (m, 16H, CH_2_-O), 4.56 (s, 2H, CH_2_-OH), 5.18 (s, 4H, CH_2_-O), 5.21 (s, 8H, Py-CH_2_-O), 6.20 (br, 3H, Ar), 6.41 (br, 6H, Ar), 7.96–8.37 (m, 36H, Py). ^13^C-NMR (CDCl_3_), δ (ppm): 29.6 (CH_2_), 61.8 (CH_2_-O), 64.5 (CH_2_-O), 66.5 (CH_2_-O), 69.3 (CH_2_-O), 71.8 (Py-CH_2_-O), 100.4 (HC_Ar_), 104.7 (HC_Ar_), 105.3 (HC_Ar_), 105.7 (HC_Ar_), 123.1–129.3 (C_py_), 130.7 (C_ipso_), 131.2 (C_ipso_), 131.3 (C_ipso_), 160.1 (C_ipso_). MALDI-TOF MS (*m/z*): 1472 (M). Calc. for C_101_H_84_O_11_: C, 82.31; H, 5.75 (%); found: C, 82.33; H, 5.75 (%).

#### 3.2.2. General Procedure

A solution of pyridine (6.1 mmol) and SOCl_2_ (6.1 mmol) in dry CH_2_Cl_2_ (100 mL) was added to **6**, **7**, **10** or **11** (6 mmol), and then this mixture was cooled to 0 °C. The reaction was carried out in nitrogen atmosphere for 7 h. After this, the solvent was evaporated and the resulting oil was dried and purified by silica gel chromatography using a mixture of hexane-dichloromethane 2:1 as an eluent.

*1,1′-(((5-(Chloromethyl)-1,3-phenylene)bis(oxy))bis(methylene))dipyrene* (**8**): Yellow powder, yield 5.28 mmol (88%). FTIR (KBr pellet, cm^−1^): 3442, 3043, 1593, 1463, 1381, 1322, 1172, 1043, 844, 712. UV-vis (CHCl_3_, nm) λ_max_: 344, 335, 328, 318, 314, 293, 276, 270, 265, 259, 242, 219, 204. ^1^H-NMR (CDCl_3_ + DMSO-d_6_), δ (ppm): 4.60 (s, 2H, CH_2_-Cl), 5.76 (s, 4H, Py-CH_2_-O), 6.84 (s, 3H, Ar, *J* = 2.2 Hz), 8.00-8.33 (m, 18H, H_Py_). ^13^C-NMR (CDCl_3_), δ (ppm): 45.7 (Ar-CH_2_-Cl), 68.3 (Py-CH_2_-O), 101.5 (C_Ar_), 107.4 (C_Ar_), 122.4 (C_py_), 124.1 (C_py_), 124.9 (C_py_), 125.6 (C_py_), 126.5 (C_py_), 126.8 (C_py_), 127.1 (C_py_), 127.5 (C_py_), 128.9 (C_py_), 130.0 (C_py_), 130.5 (C_py_), 130.9 (C_ipso_ Py), 139.2 (C_ipso_ Ar), 159.6 (C_ipso_ Ar). MS (*m/z*): 586 [M]^+^. Calc. for C_41_H_27_ClO_2_: C, 83.88; H, 4.64 (%); Found: C, 83.86; H, 4.67 (%).

*1,1′-(((((5-(Chloromethyl)-1,3-phenylene)bis(oxy))bis(propane-3,1-diyl))bis(oxy))bis(methylene))di-pyrene* (**9**): Yellow powder, yield 4.80 mmol (80%). FTIR (KBr pellet, cm^−1^): 3350, 3038, 2946, 2857, 1599, 1497, 1165, 1088, 1054, 844, 704. UV-vis (CH_3_OH, nm) λ_max_: 341, 333, 326, 292, 275, 251, 241, 217, 207. ^1^H-NMR (CDCl_3_) δ (ppm): 2.03 (m, 4H, CH_2_), 3.73 (t, 4H, CH_2_-O, *J* = 6.0 Hz), 3.90 (t, 4H, CH_2_-O, *J* = 6.0 Hz), 4.35 (s, 2H, Ar-CH_2_-OH), 5.15 (s, 4H, Py-CH_2_-O), 6.11 (t, 1H, Ar, *J* = 2.2 Hz), 6.21 (d, 2H, Ar, J = 2.2 Hz), 7.88–8.27 (m, 18H, Py). ^13^C-NMR (CDCl_3_) δ (ppm): 29.6 (CH_2_), 46.3 (Ar-CH_2_-Cl), 64.4 (CH_2_-O), 65.1 (CH_2_-O), 71.6 (Py-CH_2_-O), 100.0 (H-C_Ar_), 104.7 (H-C_Ar_), 123.4–130.7 (C_pireno_), 131.31 (C_ipso_ py), 142.86 (C_ipso_ Ar), 160.01 (C_ipso_ Ar). MS (*m/z*): 702 (M). Calc. for C_47_H_39_ClO_4_: C, 80.27; H, 5.59 (%); found: C, 80.25; H, 5.58 (%).

*1,1′,1″,1‴-((((((5-(Chloromethyl)-1,3-phenylene)bis(oxy))bis(methylene))bis(benzene-5,3,1-triyl))tetrakis(oxy))tetrakis(methylene))tetrapyrene* (**12**): Yellow powder, yield 2.52 mmol (42%). FTIR (KBr pellet, cm^−1^): 3443, 3041, 1596, 1466, 1378, 1321, 1170, 1043, 844, 711. UV-vis (CHCl_3_, nm) λ_max_: 344, 335, 328, 318, 314, 293, 276, 270, 265, 259, 242, 219, 204. ^1^H-NMR (CDCl_3_ + DMSO-d_6_), δ (ppm): 4.61 (s, 2H, CH_2_-Cl), 5.75 (s, 12H, Py-CH_2_-O), 6.83 (s, 9H, Ar, *J* = 2.2 Hz), 8.01-8.35 (m, 36H, H_Py_). ^13^C-NMR (CDCl_3_), δ (ppm): 45.7 (Ar-CH_2_-Cl), 68.3 (Py-CH_2_-O), 101.5 (C_Ar_), 107.4 (C_Ar_), 122.4 (C_py_), 124.1 (C_py_), 124.9 (C_py_), 125.6 (C_py_), 126.5 (C_py_), 126.8 (C_py_), 127.1 (C_py_), 127.5 (C_py_), 128.9 (C_py_), 130.0 (C_py_), 130.5 (C_py_), 130.9 (C_ipso_ Py), 139.2 (C_ipso_ Ar), 159.6 (C_ipso_ Ar). MS (*m/z*): 586 [M]^+^. Calc. for C_89_H_59_ClO_6_: C, 84.85; H, 4.72 (%); Found: C, 84.87; H, 4.69 (%). 

*1,1′,1″,1‴-((((((((5-(Chloromethyl)-1,3-phenylene)bis(oxy))bis(methylene))bis(benzene-5,3,1-triyl))tetrakis(oxy))tetrakis(propane-3,1-diyl))tetrakis(oxy)) tetrakis(methylene)) tetrapyrene* (**13**): Yellow powder, yield 4.0 mmol (67%): FTIR (KBr pellet, cm^−1^): 3206, 3129, 3049, 2937, 2863, 2082, 1970, 1732, 1599, 1528, 1479, 1329, 1295, 1162, 1055, 845, 752, 680. UV-vis (CH_2_Cl_2_, nm) λ_max_: 345, 329, 278, 244. ^1^H-NMR (CDCl_3_) δ (ppm): 2.01 (m, 8H, CH_2_), 3.77 (m, 16H, CH_2_-O), 4.25 (s, 2H, CH_2_-Cl), 5.16 (s, 4H, CH_2_-O), 5.20 (s, 8H, Py-CH_2_-O), 6.21 (br, 3H, Ar), 6.42 (br, 6H, Ar), 7.94-8.33 (m, 36H, Py). ^13^C-NMR (CDCl_3_), δ (ppm): 29.4 (CH_2_), 46.4 (CH_2_-Cl), 64.6 (CH_2_-O), 66.7 (CH_2_-O), 69.4 (CH_2_-O), 71.7 (Py-CH_2_-O), 100.3 (HC_Ar_), 104.6 (HC_Ar_), 105.3 (HC_Ar_), 105.7 (HC_Ar_), 123.3-129.5 (C_py_), 130.5 (C_ipso_), 131.1 (C_ipso_), 131.4 (C_ipso_), 160.2 (C_ipso_). MALDI-TOF MS (*m/z*): 1490 (M). Calc. for C_101_H_83_ClO_10_: C, 81.30; H, 5.61 (%); found: C, 81.33; H, 5.60 (%).

#### 3.2.3. General Procedure

A mixture of compound **8**, **9**, **12** or compound **13** (5.4 mmol) and cesium carbonate (10.1 mmol) in DMF (20 mL) was stirred vigorously in nitrogen atmosphere for 30 min. The porphyrin **14** (2.7 mmol) dissolved in DMF (10 mL) was added dropwise and the reaction was continued for 3 days. The mixture was filtered. The filtrate was evaporated to dryness under reduced pressure. The organic layer was dried and evaporated to dryness and purified by silica gel chromatography using a mixture of hexane-ethyl acetate 2:1 as an eluent.

*Dendrimer* **15**: Brown powder, yield 0.26 mmol (19%). FTI. (KBr pellet, cm^−1^): 3313, 3037, 2953, 2869, 1601, 1504, 1467, 1263, 1172, 1007, 842, 800, 707. UV-vis (DMF, nm) λ_max_: 329, 345, 423,518, 557, 597, 653. ^1^H-NMR (CDCl_3_), δ (ppm): −2.18 (br, 2H, NH), 5.39 (s, 8H, CH_2_-Ar), 5.92 (s, 16H, CH_2_-Py), 6.22 (br, 4H, Ar), 6.42 (br, 8H, Ar), 7.79 (d, 8H, Ar, *J* = 8.4 Hz), 7.97–8.43 (m, 72H, Py), 8.62 (d, 8H, Ar, *J* = 8.4 Hz), 8.97 (br, 8H, Porph). ^13^C-NMR (THF-d_8_/DMSO-d_6_), δ (ppm): 64.4 (CH_2_-O-Ar), 68.5 (CH_2_-O-Ar), 70.6 (Py-CH_2_-O), 100.3 (HC_Ar_), 101.6 (HC_Ar_), 105.1 (HC_Ar_), 114.6(C_Ar,porph_), 122.8-130.6 (C_py_), 127.2 (C_pyrrol_), 128.1 (C_Ar,porph_), 131.3 (C_ipso_ py), 131.1 (C_ipso_), 132.4 (C_ipso_), 136.2 (C_Ar,porph_), 138.2 (C_ipso_), 141.5 (C_pyrrol_), 160.6 (C_ipso_). MALDI-TOF MS (*m/z*): 2879 (M^+^). Calc. for C_208_H_134_N_4_O_12_: C, 86.70; H, 4.69, N 1.94 (%); found: C, 86.73; H, 4.68 (%).

*Dendrimer* **16**: Brown powder, yield 0.23 mmol (17%). FTIR (KBr pellet, cm^−1^): 3359, 3194, 2921, 2852, 1730, 1658, 1466, 1271, 1167, 1136, 847, 809, 721. UV-vis (DMF, nm) λ_max_: 243, 277, 328, 344, 422, 453, 518, 551, 595, 651, 689. ^1^H-NMR (CDCl_3_), δ (ppm): −2.23 (br, 2H, NH), 2.17 (m, 16H, CH_2_), 3.57 (m, 16H, CH_2_-O), 3.79 (m, 16H, CH_2_-O), 4.77 (s, 8H, CH_2_-Ar), 4.98 (s, 16H, CH_2_-Py), 6.25 (br, 4H, Ar), 6.36 (br, 8H, Ar), 7.56 (d, 8H _porph_, *J* = 8.2 Hz), 7.74–8.02 (m, 72H, Py), 8.07 (d, 8H, Ar, *J* = 8.2 Hz), 8.79 (br, 8H, Ar). ^13^C-NMR (THF-d_8_/DMSO-d_6_), δ (ppm): 64.8 (CH_2_-O-Ar), 68.1 (CH_2_-O-Ar), 70.9 (Py-CH_2_-O), 100.4 (HC_Ar_), 101.6 (HC_Ar_), 105.5 (HC_Ar_), 114,3 (C_Ar,porph_), 122.8-130.0 (C_py_), 127.2 (C_pyrrol_), 128.5 (C_Ar,porph_), 131.2 (C_ipso_ py), 131.3 (C_ipso_), 132.6 (C_ipso_), 136.6 (C_Ar,porph_), 138.3 (C_ipso_), 141.9 (C_pyrrol_), 161.1 (C_ipso_). MALDI-TOF MS (*m/z*): 3343 (M^+^). Calc. for C_232_H_182_N_4_O_20_: C, 83.28; H, 5.48 (%); found: C, 83.25; H, 5.43, N 1.67 (%).

*Dendrimer* **17**: Black powder, yield 0.11 mmol (8%). FTIR (KBr pellet, cm^−1^): 3313, 3038, 2923, 1602, 1503, 1470, 1233, 1173, 965, 842, 799, 708. UV-vis (DMF, nm) λ_max_: 315, 329, 345, 375, 423, 519, 555, 595, 652. ^1^H-NMR (THF-d_8_/DMSO-d_6_) δ (ppm): −2.23 (br, 2H, NH), 4.63 (s, 32H, CH_2_-Py), 5.66 (br, 16H, CH_2_-O), 5.77 (br, 8H, CH_2_-O), 6.18 (br, 36H, Ar), 7.67 (d, 8H, Ar, *J* = 8.6 Hz), 7.97–8.50 (m, 144H, Py), 8.69 (d, 8H, Ar, *J* = 8.6 Hz), 8.96 (s, 8H_pyrrol_). ^13^C-NMR (THF-d_8_/DMSO-d_6_), δ (ppm): 64.6 (CH_2_-O-Ar), 68.3 (CH_2_-O-Ar), 70.6 (Py-CH_2_-O), 100.1 (HC_Ar_), 101.8 (HC_Ar_), 105.0 (HC_Ar_), 114,6 (C_Ar,porph_), 122.8-130.9 (C_py_), 127.4 (C_pyrrol_), 128.3 (C_Ar,porf_), 131.1 (C_ipso_ py), 131.3 (C_ipso_), 132.3 (C_ipso_), 136.8 (C_Ar,porph_), 138.3 (C_ipso_), 141.4 (C_pyrrol_), 160.1 (C_ipso_). MALDI-TOF MS (*m/z*): 5567 (M^+^). Calc. for C_400_H_262_N_4_O_28_: C, 86.22; H, 4.64, N 1.01 (%); found: C, 86.23; H, 4.62 (%).

*Dendrimer* **18**: Black powder, yield 0.16 mmol (12%). FTIR (KBr pellet, cm^−1^): 3312, 3039, 2925, 2867, 1901, 1716, 1595, 1460, 1291, 1235, 1164, 1063, 966, 844, 802, 709. UV-vis (DMF, nm) λ_max_: 244, 278, 329, 345, 423, 455, 519, 557, 594, 652, 693. ^1^H-NMR (CDCl_3_/CD_3_OD) δ (ppm): −2.23 (br, 2H, NH), 1.98 (br, 32H, CH_2_), 3.63 (br, 32H, CH_2_-O), 3.97 (br, 32H, CH_2_-O), 4.61 (br, 16H, CH_2_-O), 4.82 (br, 8 H, CH_2_-O), 5.17 (br, 32H, CH_2_-Py), 6.27 (br, 36H, Ar), 7.22 (br, 16H, Ar,), 7.61–8.34 (m, 144H, Py), 8.84 (br, 8H_pyrrol_). ^13^C-NMR (CDCl_3_/CD_3_OD), δ (ppm): 29.5 (CH_2_), 60.1 (CH_2_-O), 64.5 (CH_2_-O), 66.5 (CH_2_-O), 68.7 (CH_2_-O), 71.5 (Py-CH_2_-O), 98.6 (HC_Ar_), 105.8 (HC_Ar_), 107.6 (HC_Ar_), 113.1 (HC_Ar,porph_), 119.6 (C_pyrrol_), 123.2-131.1 (C_py_), 135.6 (C_ipso_), 143.4 (C_pyrrol_), 158.6 (C_ipso_), 159.9 (C_ipso_), 160.13 (C_ipso_). MALDI-TOF MS (*m/z*): 6496 (M^+^). Calc. for C_448_H_358_N_4_O_44_: C, 86.76; H, 5.55, N 0.86 (%); found: C, 86.73; H, 5.57 (%).

### 3.3. Cubic NLO-Characterization

Finally, some of the synthesized dendrons and dendrimers were also studied as active media for cubic χ^(3)^-nonlinear optical effects such as nonlinear refraction and absorption via Z-Scan measurements (in spin-coated film samples obtained from THF-based dissolutions). The experimental Z-Scan set-up was implemented using an unpolarized laser beam from a 35 mW He-Ne laser system working at 632.8 nm (THORLABS, HRR170-1) [39]. Its energy was carefully monitored and kept constant during long Z-Scan measurements. The spatial mode of the laser beam was close to Gaussian TEM_00_. The polarization plane of the He-Ne laser beam was adjusted and controlled by means of a linear polarizer mounted on a rotation stage. The polarized laser beam was focused on the sample by means of a positive lens (f = 5 cm), so that a light power density of ~8.53 × 106 W m^−2^ reached the studied sample at the focal spot. At last, the samples were mounted on a motorized translation stage (25 mm length travel in steps of 2 µm) in order to perform Z-Scan experiments within the optical focal range. A large area Si-photodetector (EOT ET-2040) was located at ~0.96 m from the focusing lens, after a 2.5 mm diameter (~20% transmittance) diaphragm-aperture. All NLO-signals captured from photo-detectors were measured with a digital oscilloscope (Tektronix TDS, 744A), and all motion systems and set-up management were automated via a LabView control program.

## 4. Conclusions

Outstanding cubic NLO-effects were particularly measured for the dendrimer-**18** based film sample via the Z-Scan technique, where a high NLO-refractive coefficient in the order of 10^−2^ esu was found. This remarkably NLO-activity is mainly due to the high content of pyrene derivatives available in its respective second generation (dendron **13**) building units. In contrast, the first generation dendron **9** and the respective dendrimer-**16** compound exhibit lower NLO-activity. Accordingly, film samples obtained from compounds **13** and **18** consistently exhibited TPA-NLO absorption effects at same experimental conditions. However, more NLO-investigations should be performed in these materials in order to further understand the electronic and thermal contributions to the cubic nonlinearities.

## Figures and Tables

*Reagents and Conditions*: (a) LiAlH_4_, THF, 0 °C, N_2_; (b) SOCl_2_, pyridine, CH_2_Cl_2_; and (c) Br(CH_2_)_3_Cl, K_2_CO_3_, acetone, reflux.

*Reagents and Conditions*: Cs_2_CO_3_/DMF, 60 °C.

**Table 1:** UV-vis absorption maxima (λ_max_) of dendrimers **15**–**18**.

Compound	λ_max_, (nm)
**15**	329, 345, 423,518, 557, 597, 653.
**17**	315, 329, 345, 375, 423, 519, 555, 595, 652
**16**	243, 277, 328, 344, 422, 453, 518, 551, 595, 651, 689
**18**	244, 278, 329, 345, 423, 455, 519, 557, 594, 652, 693

**Table 2:** Linear and cubic nonlinear optical parameters of the dendron/dendrimer-based films measured according to the Z-Scan technique (Closed aperture Z-Scan measurements, @ λ_Z-Scan_ = 633 nm, S ≈ ~18%, *Rayleigh range:* z*_0_* = 3.1 mm).

Dendron/Dendrimer Film Sample	Linear Refractive Index: *n_0_* @ λ_Z-Scan_ = 633 nm	Linear Absorption Coefficient: *α*_0_ (@ 633 nm) [m^−1^]	Sample Thickness [nm]	Δ*φ*_0_/Δ*ψ*_0_	NLO-Refractive Index: γ/*n_2_* Z-Scan @ λ = 632nm × 10^−8^ [m^2^ W^−1^]/ × 10^2^ [esu]	NLO-Absorption: *β* (TPA or SA) [×10^−2^ m W^−1^]
**Dendron 9**	1.56 ± 0.052	54,185.5	2080	0.0/0.0	0.0/0.0	0.0
**Dendron 13**	1.59 ± 0.055	117,828.2	6590	+4.2/+0.05	+0.684/+2.56	+0.162 (TPA)
**Dendrimer 16**	1.68 ± 0.047	524,837.9	124	+0.4/0.0	+2.49/+9.27	0.0
**Dendrimer 18**	1.74 ± 0.045	1,284,314	116	+3.0/+0.05	+27.7/+104	+9.15 (TPA)

**Table 3:** Crystal data and structure refinement.

Empirical formula	C_17_H_12_O
Formula weight	232.27
Temperature	298(2) K
Wavelength	0.71073 Å
Crystal system	Monoclinic
Space group	P 21/c
Unit cell dimensions	a = 19.995(3) Å
	b = 8.9672(14) Å
	c = 13.232(2) Å
Volume	2370.8(7) Å^3^
Z	8
Density (calculated)	1.301 Mg/m^3^
Absorption coefficient	0.079 mm^−1^
F(000)	976
Crystal size/shape/color	0.32 × 0.23 × 0.10 mm/Prism/Colorless
Theta range for data collection	2.04 to 25.38°
Index ranges	−24 ≤ h ≤24, −10 ≤ k ≤10, −15 ≤ l ≤15
Reflections collected	19076
Independent reflections	4355 [R(int) = 0.0904]
Completeness to theta = 25.36°	99.9%
Absorption correction	None
Refinement method	Full-matrix least-squares on F^2^
Data/restraints/parameters	4355/2/331
Goodness-of-fit on F2	0.804
Final R indices [I > 2sigma(I)]	R1 = 0.0473, wR2 = 0.0740
R indices (all data)	R1 = 0.1546, wR2 = 0.0954
Largest diff. peak and hole	0.124 and −0.092 e.Å^−3^
